# NETS^1HD^ study: development of a Hirschsprung’s disease core outcome set

**DOI:** 10.1136/archdischild-2017-312901

**Published:** 2017-08-07

**Authors:** Benjamin Saul Raywood Allin, Timothy Bradnock, Simon Kenny, Jennifer J Kurinczuk, Gregor Walker, Marian Knight

**Affiliations:** 1 National Perinatal Epidemiology Unit, University of Oxford, Headington, UK; 2 Royal Hospital for Children, Glasgow, UK; 3 Alder Hey Children’s Hospital, Liverpool, UK

**Keywords:** Hirschsprung’s Disease, Core Outcome Set, Paediatric Surgery, Gastroenterology

## Abstract

**Objective:**

The objective of this study was to develop a Hirschsprung’s disease (HD) core outcome set (COS).

**Methods:**

Candidate outcomes were identified from a systematic review and stakeholder nomination. A three-phase Delphi process and consensus meeting were used to prioritise candidate outcomes based on scores assigned by stakeholder participants using a nine-point scale. In phases two and three, participants were shown graphical representations of their panel’s scores and all panels’ scores respectively for each outcome from the previous phase. After the third phase, outcomes prioritised by two or three panels were taken forward to the consensus meeting. The COS was formed from the 10 highest scoring outcomes meeting the threshold for inclusion (≥70% 7–9 and <15% 1–3).

**Results:**

Eighty-nine stakeholders (82%) completed all three phases of the Delphi process. Seventy-four outcomes were assessed in phase one of the Delphi process, the following 10 of which met criteria for inclusion in the COS: (1) death with cause specified, (2) long-term faecal incontinence, (3) long-term voluntary bowel movements without need for enemas, or rectal or colonic irrigation, (4) long-term psychological stress for the individual with Hirschsprung’s disease, (5) long-term urinary incontinence, (6) objective score of quality of life, (7) objective score of bowel function, (8) unplanned reoperation, (9) >need for a permanent stoma, (10) enterocolitis.

**Conclusions:**

This HD COS is formed of 10 outcomes deemed important by key stakeholders. Use of this COS in research will reduce outcome reporting heterogeneity and increase our ability to identify gold standard treatments for HD.

**What is known on this topic?**

- Outcome reporting heterogeneity in published research is preventing identification of gold standard treatments for infants with Hirschsprung’s disease.
- Developing and using core outcome sets in research reduces outcome reporting heterogeneity.

**What this study adds?**

- This study has identified 10 outcomes of importance to key stakeholders including people with Hirschsprung’s disease, parents of children with Hirschsprung’s disease and healthcare professionals managing children with Hirschsprung’s disease.
- Use of this HD core outcome set will reduce outcome reporting heterogeneity, making it easier to identify gold standard treatments for children with Hirschsprung’s disease.

## Background

Hirschsprung’s disease (HD) affects 1.8 in 10 000 live-born children in the UK and Ireland and is caused by failure of complete development of the nerves of the enteric nervous system. Definitive treatment requires excision of the affected colon, with anastomosis of the remaining normal colon to the anus or rectum. Globally, there are differences in management strategies.^1^ Operative strategies include open, laparoscopic and purely transanal approaches, and the Duhamel,^2^ Swenson^3^ and Soave^4^ anastomotic techniques. Each strategy has potential advantages and disadvantages and at present it is not possible, either with primary data or through conduct of systematic reviews, to identify a gold standard approach.^5 6^ The reasons for the lack of clarity are multifactorial. Specifically, most studies are small, single-centre, observational, of short duration and retrospective,^7^ with significant heterogeneity of outcome reporting.^8^

Outcome reporting heterogeneity makes the evidence base difficult to interpret in three ways. First, it creates a risk that studies fail to address outcomes of relevance to patients, clinicians and commissioners of healthcare. Second, it suggests and increased risk of reporting bias within the published literature, and finally, it limits the conduct of meta-analyses.

Core outcome sets (COS) are groups of standardised outcomes that have been identified by key stakeholders as being the most important in determining success of an intervention or treatment of a particular condition.^9^ Once a COS has been developed for a condition, the intention is that all future studies of that condition should report data for every outcome within the COS. Development and use of COS in this manner reduces outcome reporting heterogeneity,^10^ making it easier to identify gold standard treatments. Journal editors, the National Institute for Health Research Health Technology Assessment Programme and the IDEAL collaboration have endorsed this approach (http://www.comet-initiative.org/about/COMETendorsement). The objective of this work was therefore to develop a COS that could be used in studies comparing interventions for infants with HD.

## Methods

**Figure 1:**
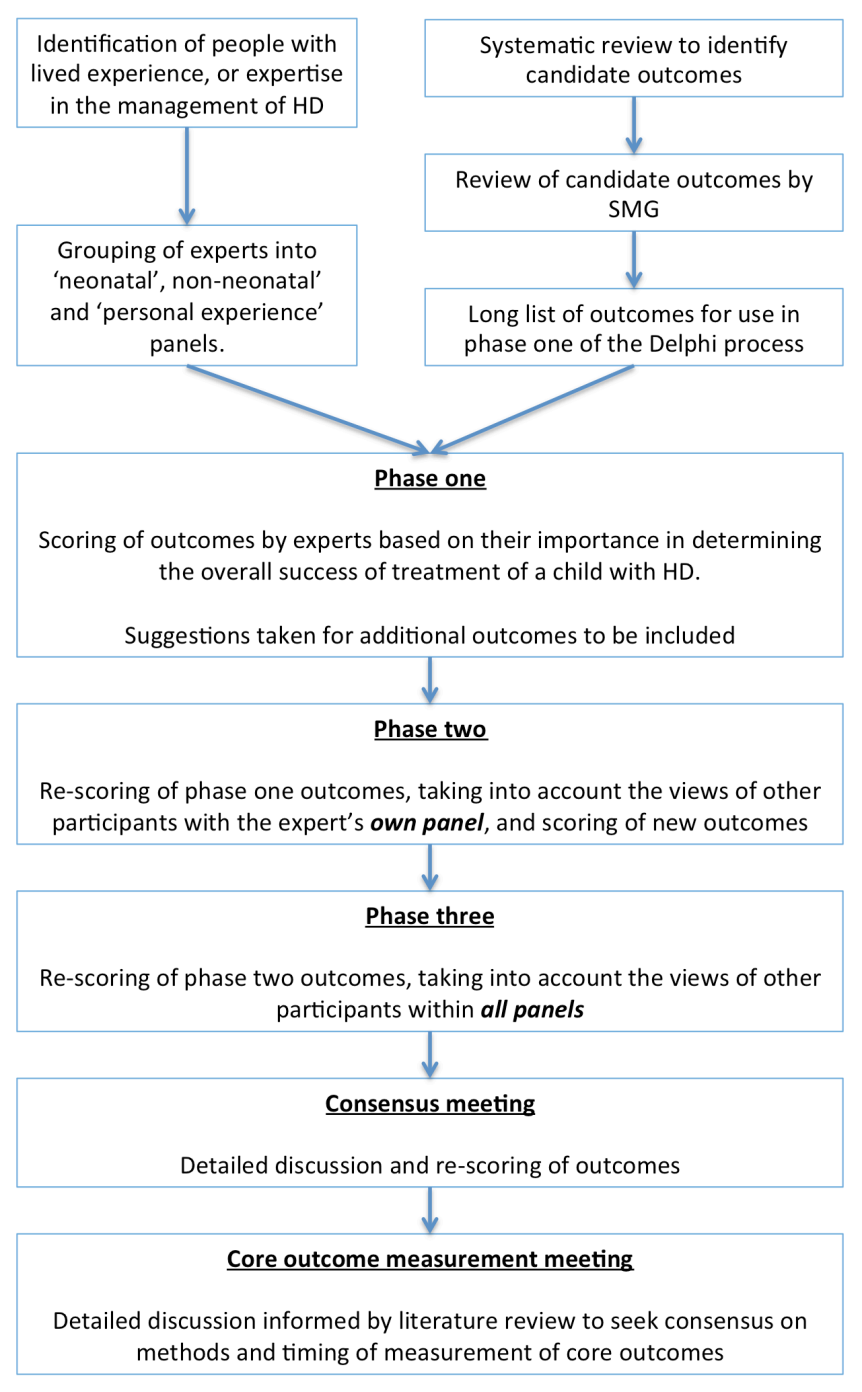
Study overview. HD, Hirschsprung’s disease; SMG, study management group.

The COS was developed according to a prospectively registered protocol,^11^ using methodology recommended by the COMET initiative (figure 1).

### Scope

The aim was to develop a COS for use in studies comparing interventions for the treatment of infants with HD in high-income countries. It may need adaptation for low-income and middle-income countries.

### Participants

Participants were recruited from key stakeholder groups with either expertise in the management of infants with HD or lived experience of HD (table 1). UK and international participants were recruited.

**Table 1:** Recruitment targets for key stakeholder groups

Stakeholder group	Panel	Recruitment targets
Paediatric surgeons	Neonatal panel	Surgeons registered with the British Association of Paediatric Surgeons (BAPS) as having an interest in HD surgery.
		Centre leads for the British Association of Paediatric Surgeons Congenital Anomalies Surveillance System (BAPS-CASS) nationwide HD cohort study.
		Members of the United Kingdom Paediatric Colorectal Club
		Editors of the *Journal of Paediatric Surgery* and *Paediatric Surgery International*
		Members of BAPS with a self-declared interest in the management of infants with HD
		Named experts from prominent HD treatment centres and research groups.
Neonatologists	Neonatal panel	Members of the British Association of Perinatal Medicine with a self-declared interest in the management of infants with HD
		Members of the Royal College of Paediatrics and Child Health with a self-declared interest in the management of infants with HD
Paediatric gastroenterologists	Non-neonatal panel	Members of the British Society of Paediatric Gastroenterology, Hepatology and Nutrition with a self-declared interest in the management of infants with HD
Specialist nurses	Non-neonatal panel	Members of the paediatric stoma nurses group
People with HD and parents of children with HD	Personal experience panel	Parents of children with HD who are members of the Parent Advisory Group established by the National Perinatal Epidemiology Unit
		Members of the Hirschsprung’s and Motility Disorders Support Network
		Members of the CHAMPS appeal HD support group

Parents of children treated by members of the SMG and members of the BAPS-CASS Steering Committee.

HD, Hirschsprung’s disease; SMG, study management group.

An iterative process^12^ was used to recruit participants across stakeholder groups, including among others, paediatric surgeons, gastroenterologists, people with HD and parents of children with HD. Members of all stakeholder groups had an equal role in the prioritisation of outcomes, so as to ensure that the final COS represented as best as possible, the views of those with expertise in managing children with HD and those with lived experience of HD. Members of the study management group (SMG) identified experts known to them, and then nominated groups from which additional experts could be recruited (table 1). Electronic media for each of the appropriate organisations were used to distribute adverts to their membership. Experts registering to participate in the study were asked to provide information relating to their experiences of HD and to nominate other potential participants. Registration details for all experts were reviewed by the SMG to ensure they had sufficient expertise to participate. Target recruitment was a minimum of 50 experts with two or more from each stakeholder group.

Participants were deemed to have withdrawn from the development process if they did not complete a phase of the Delphi process prior to the prespecified deadline, and were thus ineligible for participation in later phases or the consensus meeting. A representative sample of participants completing the Delphi process were invited to the subsequent consensus meeting and measurement meeting.

### Information sources

Candidate outcomes were identified from a systematic review of surgical interventions for the primary, definitive treatment of infants with HD.^8^ Additional outcomes of importance identified by the SMG but not identified in the systematic review were added to this list. At the end of phase one of the Delphi process, participants could suggest additional outcomes that were of importance to them, and if within the scope of the COS, these were added to phase two. Lay equivalent language for each outcome was developed in conjunction with parents without a medical or scientific background. Each outcome was assigned by the SMG to a core area of the OMERACT 2.0 filter (death, life impact, pathophysiological manifestation or resource use/economical impact) and identified as an adverse event if appropriate, using the guidelines described by Boers *et al*.^13^

### Consensus process

A three-phase online Delphi process was conducted in parallel for the three panels and was followed by a face-to-face consensus meeting.

In phase one, participants were asked to score candidate outcomes based on their importance in deciding whether the overall treatment of a child’s HD had worked well. Participants were provided with written instructions to score from 1 to 9 where 1, 2 and 3 were ‘not that important’, 4, 5 and 6 were ‘important’ and 7, 8 and 9 were ‘really important’. In phase two, participants were shown graphical and numerical representations of their panel’s median score and distribution of scores for each outcome from phase one and asked whether they would like to change their scores based on this information. In phase three, participants were shown graphical and numerical representations of all panels’ median scores and distribution of scores for each outcome, and again asked if they would like to change their scores based on this information.

### Outcome dropping and modification

Following phase two, outcomes where ≥50% of participants in any panel had scored them 1–3 and <50% of participants in all panels had scored them 7–9 were dropped. Following phase three, outcomes were deemed to meet the threshold for automatic discussion and rescoring at the consensus meeting if two or more panels deemed them to meet consensus for inclusion in the COS. Consensus for inclusion was defined as scores of ≥70% 7–9, and <15% 1–3. Other outcomes were only discussed and rescored if there was unanimous agreement among consensus meeting attendees that they warranted further review.

### Consensus definition

Outcomes with scores of ≥70% 7–9 and <15% 1–3 following discussion at the consensus meeting were eligible for inclusion in the COS. However, to ensure practicality of use, it was prespecified that 10 or fewer outcomes would be included in the COS. If more than 10 outcomes were eligible for inclusion, then only the following 10 would be included:

- the highest scoring outcome meeting consensus for inclusion in each of the four OMERACT 2.0 filter core areas
- the highest scoring adverse event outcome meeting consensus for inclusion in the COS (if not already included as the highest scoring outcome in one of the four core areas)
- the next five highest scoring outcomes meeting consensus for inclusion in the COS, regardless of OMERACT 2.0 filter core area.

As the highest scoring adverse event outcome meeting consensus for inclusion in the COS was already included as one of the highest scoring outcomes from the core areas of the OMERACT 2.0 filter; the sixth highest scoring outcome (not already included) meeting consensus for inclusion in the COS was also included.

Highest scoring was defined as greatest percentage of participants allocating scores of 7–9. Where outcomes were tied based on this score, then the highest scoring outcome was the one with the greatest percentage of participants allocating a score of 9, then 8, then 7, continued through to 1 if necessary.

### Outcome definition and measurement

A literature review was conducted to identify existing definitions and methods of measuring the outcomes included in the COS following the consensus meeting. This review informed discussion at a meeting attended by a representative sample of study participants, where outcome definition and measurement were identified by group consensus.

## Results

### Changes from protocol

The following changes to protocol were made after registration but prior to data analysis.

No dropping of outcomes between phases of the Delphi process or between the Delphi process and the consensus meeting was originally planned. However, it was decided that outcomes should be dropped as described to allow participants to focus on discriminating between those outcomes most likely to form the COS.

No limit on the number of outcomes to be included in the COS was originally planned. However, in order to ensure practicality of use, it was determined that the COS would be limited to 10 outcomes.

### Participants

One hundred and forty-five experts registered to participate in the study, 108 (74%) of whom completed phase one of the Delphi process. Ninety-six eligible participants (89%) completed phase two, and 89 eligible participants (93%) completed phase three. Seventeen participants were selected to attend the consensus meeting, and 14 attended the measurement definition meeting (tables 2 and 3).

**Table 2:** Summary of participants

	Number of participants					
	Registering for round one	Completing round one (% of those eligible)	Completing round two (% of those eligible)	Completing round three (% of those eligible)	Consensus meeting	Measurement meeting
Neonatal panel	41	34 (83)	33 (97)	31 (94)	9	7
Non-neonatal panel	15	13 (87)	12 (92)	12 (100)	2	3
Personal experience panel	89	61 (69)	51 (84)	46 (87)	6	4
Total	145	108 (74)	96 (89)	89 (93)	17	14

**Table 3:** Characteristics of participants who completed the Delphi process and those who dropped out of the study

**Parents of children with HD**												
	Location* (n (%))		**Age of child (years) (n (%))**				**Parent-reported severity of child’s HD‡ (n (%))**				**Gender of participant (n (%))**	
	**UK**	**Other**	**≤5**	**6–10**	**11–15**	**≥16**	**Median on 1–9 scale (IQR)**	**Ultra-short segment**	**Short segment**	**Long segment**	**Male**	**Female**
Completed Delphi process	29 (76)	9 (24)	19 (49)	13 (33)	1 (3)	6 (15)	7 (6–8)	1 (3)	22 (59)	14 (38)	1 (3)	38 (97)
Only completed phase one	12 (86)	2 (14)	6 (43)	5 (36)	1 (7)	2 (14)	Not collected until phase two				14	0
**People with HD**												
	**Location (n (%))**		**Age (n (%))**				**Self-reported severity HD^§^ (n (%))**				**Gender (n (%))**	
	**UK**	**Other**	**11–15 years old**		**≥21 years old**		**Median on 1–9 scale (IQR)**	**Ultra-short segment**	**Short segment**	**Long segment**	**Male**	**Female**
Completed Delphi process	4 (57)	3 (43)	1 (14)		6 (86)		7 (5–8)	0 (0)	0 (0)	6 (100)	4 (57)	3 (43)
Only completed phase one	1 (100)	0 (0)	0 (0)		1 (100)		Not collected until phase two				0 (0)	1 (100)
**Neonatal panel**												
	**Location (n (%))**		**Prior involvement in HD research (n (%))**		**Number of HD patients treated per year (n (%))**		**Stakeholder group (n (%))**				**Gender (n (%))**	
	**UK**	**Other**	**No**	**Yes**	**≤10**	**>10**	**Paediatric surgeons**		**Neonatologists**		**Male**	**Female**
Completed Delphi process	29 (94)	2 (6)	11 (35)	20 (65)	21 (68)	10 (32)	27 (87)		4 (13)		23 (74)	8 (26)
Only completed phase one	2 (66)	1 (33)	0 (0)	3 (100)	0 (0)	3 (100)	3 (100)		0 (0)		3	0
**Non-neonatal panel**												
	**Location (n (%))**		**Prior involvement in HD research (n (%))**		**Number of HD patients treated per year^†^ (n (%))**		**Stakeholder group (n (%))**				**Gender (n (%))**	
	**UK**	**Other**	**No**	**Yes**	**≤10**	**>10**	**Specialist nurses**	**Paediatric gastroenterologists**		**HD Researchers**	**Male**	**Female**
Completed Delphi process	12 (100)	0 (0)	9 (75)	3 (25)	7 (58)	3 (42)	7 (58)	4 (33)		1 (8)	4 (33)	8 (66)
Only completed phase one	1 (100)	0 (0)	1 (100)	0 (0)	1 (100)	0 (0)	0 (0)	1 (100)		0 (0)	1 (100)	0 (0)

*One person preferred not to say.

† One preferred not to say, one not applicable.

‡ One person preferred not to score, and two did not know the length of affected bowel.

§ One person did not know the length of affected bowel.

HD, Hirschsprung’s disease.

### Outcomes: initial phase one list

Seventy-four outcomes were identified by the systematic review, nine were added by the SMG, and nine were excluded as outside of the scope of the COS (online supplementary material 1).

10.1136/archdischild-2017-312901.supp1 Supplementary material 1

### Outcomes: addition, dropping and formation of the COS

**Figure 2:**
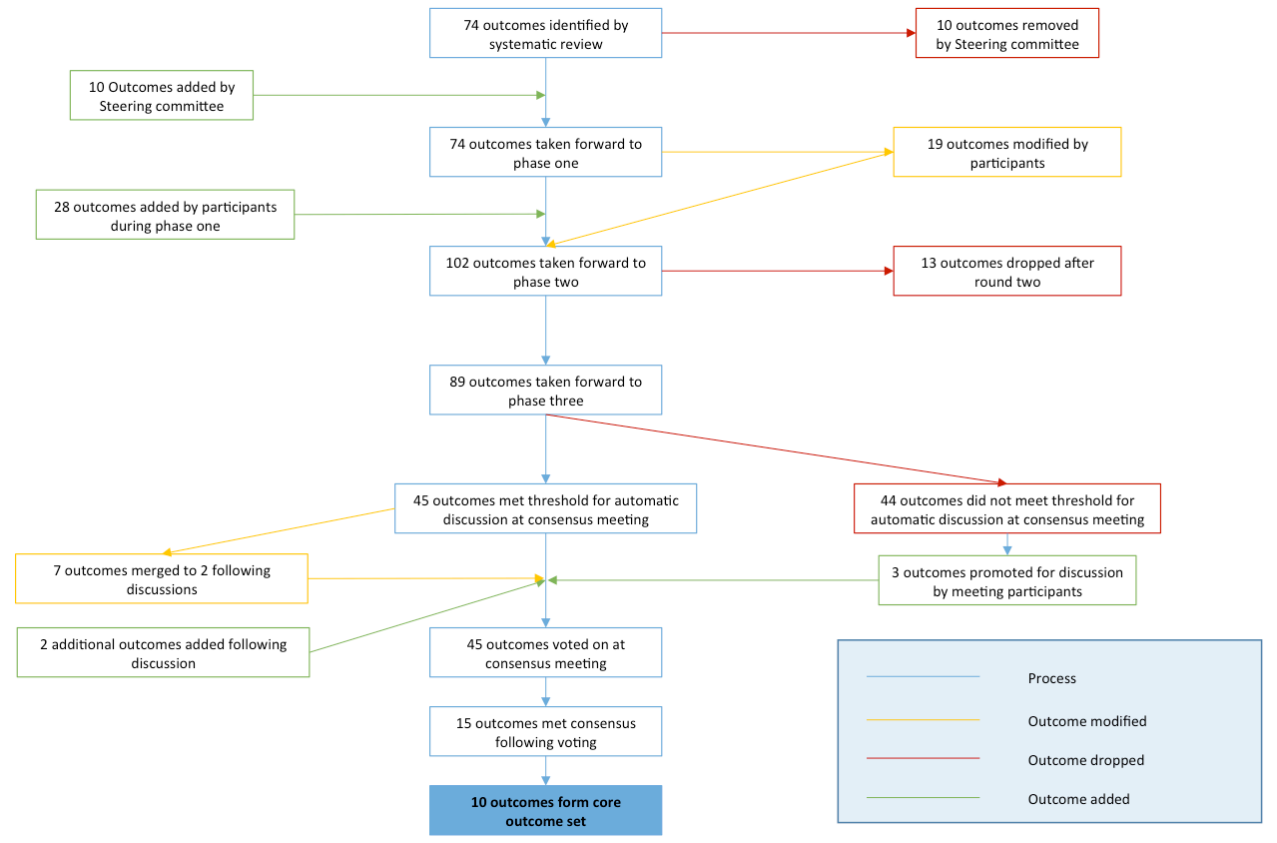
Outcome flow.

Overall 140 individual comments were made by participants during phase one, 62 of which (44%) were made by 15 members (44%) of the neonatal panel, 73 (52%) by 35 members (57%) of the personal experience panel and 5 (4%) by 2 members (15%) of the non-neonatal panel. Based on these comments, the SMG clarified 19 outcomes (26%) and added 28 new outcomes for assessment in phase two. Six of these outcomes (21%) were proposed by the personal experience panel, 6 (21%) by other panels, and the remaining 16 (57%) by multiple panels. Overall, 102 outcomes were taken forward to phase two. Following completion of phase two, 13 outcomes (13%) were dropped, and following phase three, 44 outcomes (49%) did not meet criteria for automatic progression to the consensus meeting. Forty-five outcomes were taken to the consensus meeting, following which 15 outcomes met the criteria for inclusion in the COS, and 10 were retained (figure 2, tables 4 and 5 and online supplementary material S1). These 10 core outcomes are described in table 5.

**Table 4:** Outcomes scored at the consensus meeting

Mortality outcomes	Resource utilisation outcomes	Life impact outcomes		Pathophysiological manifestation outcomes	Adverse event outcomes	
Death with cause specified*	Whether home parenteral nutrition is required	Long-term faecal incontinence*	Impotence	Aganglionic bowel remaining at the proximal resection margin	Intraoperative complication—life threatening (Clavien-Dindo grade four)	Postoperative bowel obstruction
	Unplanned reoperation with indication specified*	Long-term voluntary bowel movements without need for enemas, or rectal or colonic irrigation*	Difficulty in conceiving a child	Hirschsprung’s associated enterocolitis*	Postoperative complication—life threatening (Clavien-Dindo grade four)	Narrowing of anastomosis
	Unplanned readmission	Long-term urinary incontinence*	Attendance at school, or time spent missing lessons	Normal growth	Intraoperative visceral injury—life threatening (Clavien-Dindo grade four)	Fistula
	Associated healthcare costs	Long-term offensive odour secondary to lack of control of faeces or flatus, or inability to maintain hygiene	Long-term psychological stress-family*	Objective score of bowel function*	Anastomotic leak	Any intraoperative visceral injury
	Societal costs*	Requiring nappy or pad	Long-term bladder dysfunction		Colonic torsion	Cuff infection
		Sensation of need to defecate*	Painful defecation		Ischaemic bowel	Wound dehiscence
		Perianal excoriation with significant impact on daily life*	Difficulty with sexual relations or sexual intercourse because of the psychological or physical impact of HD or its treatment*		Faecal impaction	
		Peristomal excoriation with significant impact on daily life	Long-term difficulty in feeding			
		Need for a permanent stoma, with indication specified*	Social development			
		Need for a new stoma at any point after the pull-through procedure	Objective score of quality of life, using appropriate age specific measures*			
		Long-term psychological stress for the individual with Hirschsprung’s disease*	Urgency of stool			

*Outcomes meeting consensus for inclusion in the COS.

COS, core outcome set.

**Table 5:** The Hirschsprung’s disease core outcome set

Core outcome	Core area	Definition	Score 7–9 (%)	Minimum age of measurement
**Highest scoring outcomes in each OMERACT Filter 2.0 core area**				
**Death with cause specified**	Death	Death, with cause classified as due to A complication of treatment (excluding Hirschsprung-associated enterocolitis) Hirschsprung-associated enterocolitis An associated anomaly Other.	100%	No minimum
**Long-term faecal incontinence**	Life impact	Involuntary passage of faecal matter in an inappropriate place by a child aged 5 years or over. Severity of faecal incontinence should be graded as:* Occasionally (eg, once or twice per week), with or without social problems Every day, but without social problems Constant, with social problems.	100%	Five years of age
**Objective score of bowel function**	Pathophysiological manifestations	Objective score of bowel function, as measured by the Paediatric Incontinence and Constipation Score in children under 18 years of age, and the Gastrointestinal Quality of Life Index in adults over 18 years of age.	94%	No minimum
**Unplanned reoperation, with indication specified**	Resource use/economical impact and adverse event category	Unplanned reoperation with indication classified according to NICE criteria as minor, intermediate or major/complex. Unplanned is defined as any procedure not considered part of routine post-intervention practice. This outcome should include any procedure that is performed as a direct result of the diagnosis or treatment of the participants HD, and any episodes of general anaesthesia that are required as a direct result of the diagnosis or treatment of the participant’s HD, regardless of whether an operative intervention is undertaken (eg, an examination under anaesthesia, or manual evacuation).	89%	No minimum

**Core outcome**	Definition	Score 7–9 (%)	Minimum age of measurement
**Outcomes included regardless of OMERACT core area**			
**Long-term voluntary bowel movements without need for enemas, or rectal of colonic irrigation**	Long-term voluntary bowel movements without need for enemas, or rectal or colonic irrigation.	100%	Five years of age
**Long-term psychological stress for the individual with HD**	Long-term psychological stress for the individual with HD as measured by the PedsQL in children under 18 years of age, and the Gastrointestinal Quality of Life index in adults over the 18 years of age.	100%	Five years of age
**Long-term urinary incontinence**	Involuntary voiding of urine that is constant, associated with social problems or requiring catheterisation.	94%	Five years of age
**Objective score of quality of life using appropriate age-specific measures**	Quality of life as measured by the age-appropriate PedsQL questionnaire.	94%	Five years of age
**Need for a permanent stoma, with indication specified**	Need for permanent stoma as a direct result of the diagnosis, or treatment of the participant’s HD, including where the decision for a stoma has been made out of patient, or parental preference, or for continence management. The indication for stoma formation should be reported. A permanent stoma is defined as one which was created without the intention of later reversal.	88%	Five years of age
**Hirschsprung-associated enterocolitis**	A score of 10 or more on the Hirschsprung-associated enterocolitis Delphi score developed by Pastor *et al*. Where it is not possible to use the Hirschsprung-associated Delphi score, for example, in hospitals where left shift of white cells is not reported, Hirschsprung-associated enterocolitis should instead be defined as ‘Clinician decision to admit and instigate treatment for Hirschsprung’s Associated Enterocolitis’. Information should be reported on whether a participant has had any episodes of Hirschsprung-associated enterocolitis up to a standard time-point, but also whether they have had 0, 1 or 2 episodes of Hirschsprung’s associated enterocolitis in the previous 12 months, or up to the time-point of measurement, whichever is shorter.	88%	No minimum

*Based on a modification of the Krickenbeck classification^18^

†Long-term psychological stress for the individual with HD, and objective score of quality of life using appropriate age-specific measures were both identified as outcomes that should be included in the core outcome set. However, following an extensive review of the existing literature and discussion at the measurement meeting, consensus was reached that the most appropriate way to measure both outcomes would be with the PedsQL questionnaire. Therefore, both outcomes can be incorporated in the same measure in studies utilising this Hirschsprung’s disease core outcome set.

HD, Hirschsprung’s disease; PEDsQL, Pediatric Quality of Life Inventory.

### Proposed timing and measurement of outcomes

Consensus from meeting attendees was that outcomes should be measured at standard surgical and paediatric time-points determined by study design. For studies where infants entered at the point of surgical intervention, these were defined as 30 days, 90 days, 1 year, 5 years, 10 years and every subsequent 10 years postintervention. For studies where infants entered at a set age, outcome measurement points were defined as 28 days of age, 1 year of age, 5 years of age, 10 years of age and every subsequent 10 years. It was agreed that six outcomes (table 5) should not be reported prior to 5 years of age, as early data on these outcomes are likely to be misleading.

## Discussion

This development process identified a COS consisting of 10 items for use in studies comparing interventions for the treatment of infants with HD. These outcomes represent factors important to stakeholders and span the breadth of the OMERACT filter 2.0. When used in appropriate studies, they will provide a rounded assessment of different interventions for HD. All studies comparing interventions for the treatment of children with HD should report data at the specified time-points for the outcomes within the COS.

We believe this to be the first paediatric general surgical COS. However, paediatric COS have previously been developed, including for asthma and otitis media in children with cleft palate.^14 15^ Some common themes emerge from all three, including the prevalence of factors relating to quality of life, which are likely to be common to many paediatric COS, but which are currently infrequently investigated.^8^ The COIN study (http://www.comet-initiative.org/studies/details/842?result=true) will develop a COS for neonatology, and the NETS^1G^ study^16^ is developing a COS for gastroschisis. Following completion of these, it will be important to compare and contrast the outcomes of importance in each so as to identify areas of overlap from which a unified neonatal surgical COS could potentially be developed.

This HD COS has been developed using robust methodology in accordance with recommendations from the COMET initiative. Participation rates were significantly above our target recruitment of 50 experts, with a good spread across stakeholder groups and good retention throughout the process. We therefore believe the COS to be representative of the views of the HD community as a whole. Increasingly however, there is a move towards undertaking qualitative work with key stakeholders in addition to a systematic review to inform the long-list of outcomes assessed during the Delphi process. This methodology is being promoted as there is a suspicion that outcomes identified through systematic reviews may be biased in favour of clinicians and researchers.^17^

Instead of using qualitative methods to inform the Delphi process, we opted to conduct the Delphi in an adaptive manner. All participants were asked to recommend additional outcomes of importance to them at the end of phase one and could suggest modifications to outcomes at all stages of the consensus process. The low number of additional outcomes proposed by the personal experience panel suggests that either they felt the initial list covered the majority of outcomes of importance to them or that they felt unable to propose additional outcomes. As over 50% of comments made in phase one were from members of the personal experience panel, and nearly 60% of the personal experience panel made at least one comment during phase one, we believe the former theory to be more likely. It is also reassuring that there was similarity in the number of outcomes suggested by each panel and that there was overlap in the domains from which additional outcomes were suggested by each panel.

There are three areas that may affect the representativeness of the COS. First, from the personal experience panel, there was a larger proportion of women taking part than men, meaning that the views of fathers, and men with HD, are potentially under-represented. Second, there was a slightly greater proportion of participants with long-segment and ultra-short segment HD within the study population than would be expected within the general population. This may skew the priorities of the personal experience panel more towards the extremes of the disease population. Third, the non-neonatal panel consisted of fewer participants than either the neonatal or personal experience panels. By giving equal weight to each panel as opposed to each individual participant throughout the Delphi process, individual participants within the non-neonatal panel will have had a proportionately greater influence on the scoring of outcomes throughout the Delphi process than participants in other panels. At the consensus meeting however, the meeting attendees were treated as one group, not individual panels. Within this setting, therefore, where there were less experts from the non-neonatal panel than from other panels, there is potential for the views of members of the non-neonatal panel to have been under-represented in the final COS.

This study has incorporated the views of key stakeholders to develop a COS for use in studies conducted in high-income countries comparing interventions for the treatment of infants with HD. It is important to promote the use of this COS in future large-scale observational and interventional studies but also in smaller, retrospective studies that still comprise the vast majority of paediatric surgical research.^7^ Doing so will ensure studies are relevant to patients and their family, reduce the risk of reporting bias and, importantly, make meta-analysis possible. In the long run, this will improve the evidence base used to support clinical management of infants born with HD and should eventually allow translation through into improvements in patient care. It is now incumbent on funding bodies, journal editors and key decision makers in the field of paediatric surgical research to ensure that the COS is widely implemented and the benefits of its use realised.
