# Supplementary material for: NETS1HD study: development of a Hirschsprung’s disease core outcome set
Source: Arch Dis Child. 2017 Aug 7;102(12):1143–51. doi: 10.1136/archdischild-2017-312901 (PMC5754863; doi:10.1136/archdischild-2017-312901)
Supplement: Supplementary material 1 [file archdischild-2017-312901supp001.docx]

## Supplementary material one: Outcomes taken forward to phase one of the NETS^1HD^ Delphi process

Following review of the 74 unique outcomes identified by the systematic review, the SMG determined that there were four outcomes (5%) that fell outside of the scope of the COS, and so these were not taken forward to phase one of the Delphi process. There were nine outcomes (12%) relating to the areas of bowel function, bowel obstruction, and feeding that overlapped significantly, and hence were merged to four common outcomes. Three outcomes (4%) relating to the areas of perianal excoriation, re-admission, and anastomotic narrowing were each felt to represent multiple outcomes, and so were split by the SMG into seven separate outcomes. There were three outcomes (4%) where definitions were clarified by the SMG prior to phase one of the Delphi process. The SMG felt there were an additional five outcomes that may be important, but which had not been identified by the systematic review. These were added to the long list of outcomes prior to phase one of the Delphi process. In total, nine outcomes were removed, and nine were added, meaning the number of outcomes taken forward to phase one of the Delphi process remained at 74 , and are shown in the table below.

| **Professional Term** | **Lay Term** |
| --- | --- |
| Abdominal distension | Whether the tummy remains swollen/distended |
| Abscesses | Whether an abscess (a collection of pus) develops after the operation |
| Adhesions | Whether adhesions (scar tissue) form inside the abdomen |
| Aganglionic bowel remaining at the proximal resection margin | Whether there is any abnormal bowel remaining after the operation. |
| Analgesic use post-operatively | How much pain relief was needed after the operation |
| Anastomotic Leak | Whether the point where two bits of bowel have been joined together (anastomosis) develops a leak |
| Anorectal Manometry | What pressure is produced by the muscles that control when the bowels open (as measured by a test called anal manometry) |
| Bladder Dysfunction | Whether the bladder works normally |
| Colonic torsion | Whether the bowel was accidentally left twisted at the end of the operation |
| Consistency of stool | How hard or soft the poo is |
| Conversion to open | Whether an operation that started as a keyhole operation has to instead be performed through a bigger wound |
| Cosmetic results | What the tummy looks like after the operation, for example the appearance of the scars |
| Cost | How much the operation cost |
| Cuff infection | Whether there is an infection of the layer of muscle around the bowel where it has been joined onto the bottom. |
| Death | Death |
| Dehiscence | Whether the cuts that were made to do the operation open up again |
| Depression | Depression |
| Dilations | Whether regular stretching of the anus is required |
| Duration of post-operative antibiotic use | How long antibiotics were given for after the operation |
| Early pelvic inflammation | Whether there is internal inflammation (swelling) after the operation |
| Enterocolitis | Development of Hirschsprung's Enterocolitis (a serious infection of the bowel) |
| Faecal Impaction | When hard poo gets stuck in the bowel, needing to be cleared by medicines, enemas (liquid medicine given via the bottom), or manually removed |
| Faecal incontinence | Unable to control the bowels so poo leaks out without control |
| Fever | Whether there is an abnormally high temperature after the operation |
| First post-operative bowel movement | How long it was between the operation and bowels first opening |
| Fistula | Whether an abnormal connection called a fistula is formed between the skin and part of the bowel |
| Frequency of bowel movements | How often the bowels open |
| Granuloma | Abnormal excessive healing tissue around a wound |
| Hospital stay (length) | How long the total hospital stay was after the operation |
| ICU or HDU admission | Whether admission to the intensive care unit or high dependency unit was needed after the operation |
| Impotence | Inability to achieve or maintain an erection |
| Incisional hernia | Whether an incisional hernia develops. An incisional hernia is where there is a weakness in the muscle under the cuts that were used to perform the operation, so that that bowel pokes through the muscle causing a lump under the skin. |
| Intra-operative blood loss | Whether there is bleeding during the operation |
| Intra-operative complication | Whether there is a complication during the operation |
| Intra-operative visceral injury | Whether there is injury to another organ during the operation |
| Ischaemic bowel | Whether the blood supply to the bowel is affected during the operation |
| Laxative medications | Whether laxatives (medicines that make the poo softer, or increase how often the bowels open) are required |
| Long-term difficulty in feeding, or intolerance of food | Whether there is difficulty in feeding |
| Narrowing of anastomosis | Whether there is a narrowing of the point where healthy bowel has been joined to the bottom |
| Narrowing of cuff | Whether the layer of muscle around the bowel where it has been joined to the bottom becomes too tight |
| Objective score of bowel function | A scoring system that gives an overall numerical representation of how well the bowels are working |
| Objective score of quality of life | A scoring system that gives an overall numerical representation of how good quality of life is |
| Operation length | How long the operation took |
| Painful defecation | Whether it is painful to go for a poo |
| Parastomal Hernia | Development of a hernia around the stoma. A stoma is where the bowel is joined to the skin on the tummy wall so that poo goes into a bag on the tummy as opposed to through the bottom. A hernia is where there is a weakness in the muscle so that that bowel pokes through the muscle causing a lump under the skin. |
| Parenteral nutrition use | Whether nutrition given through a drip into a vein is required (intravenous nutrition, or TPN) |
| Peri-anal Excoriation with significant impact on daily life | Whether there is nappy rash (irritation and breakdown of the skin around the bottom), that is having a significant impact on daily life, e.g. requiring extensive skin care regimes |
| Peri-anal Excoriation without significant impact on daily life | Whether there is nappy rash (irritation and breakdown of the skin around the bottom), without significant impact on daily life |
| Peri-stomal Excoriation with significant impact on daily life | Irritation and breakdown of skin around the stoma that has a significant impact on daily life e.g. requiring excessive stoma bag changes, or extensive skin care regimes. A stoma is where the bowel is joined to the skin on the tummy wall so that poo goes into a bag on the tummy as opposed to through the bottom. |
| Peri-stomal Excoriation without significant impact on daily life | Irritation and breakdown of skin around the stoma that has a minimal impact on daily life. A stoma is where the bowel is joined to the skin on the tummy wall so that poo goes into a bag on the tummy as opposed to through the bottom. |
| Planned readmission (e.g. for anal dilatations) | Whether another planned admission was needed after being discharged from the initial operation e.g. for regular stretching of the anus |
| Pneumonia | Whether a chest infection develops after the operation |
| Post-operative 'complications' | Whether there are complications after the operation |
| Post-operative Bowel obstruction | Whether the bowel becomes blocked after the operation |
| Post-operative experience of pain | How much pain was experienced after the operation |
| Post-operative infection | Whether there is an infection after the operation |
| Psychological stress | Stress and Anxiety |
| Reoperations | Whether more than one operation is required |
| Requiring nappy or pad | Whether a nappy or pad is required |
| Sensation of need to defecate | Whether there is the normal feeling of needing to open the bowels |
| Sleep disturbance | Sleep disturbance |
| Social development | Development of normal social interactions |
| Sphincter achalasia | High pressure of the muscles around the bottom that control when the bowels open (as measured by a test called anal manometry) |
| Stoma morbidity | Whether there are problems with the stoma (if one was made). A stoma is where the bowel is joined to the skin on the tummy wall so that poo goes into a bag on the tummy as opposed to through the bottom |
| Stomal Prolapse | Whether the stoma comes out further than it should. A stoma is where the bowel is joined to the skin on the tummy wall so that poo goes into a bag on the tummy as opposed to through the bottom |
| Stomal retraction | Whether the stoma shrinks back inside the abdomen. A stoma is where the bowel is joined to the skin on the tummy wall so that poo goes into a bag on the tummy as opposed to through the bottom |
| Stomal stenosis | Whether the stoma becomes narrowed. A stoma is where the bowel is joined to the skin on the tummy wall so that poo goes into a bag on the tummy as opposed to through the bottom |
| Time to full feeds (post-operatively) | Time between the operation and taking full feeds by mouth without any need for fluid or feed through a drip |
| Time to normal bowel habits | How long there is between the operation and bowels working normally |
| Time until first feed (post-operatively) | Time between the operation and first being able to have milk/food by mouth |
| Unplanned Readmission | Whether another unplanned admission was needed after being discharged from the initial operation |
| Urgency of stool | Whether it is difficult to get to the toilet for a poo in time without an accident |
| Urinary incontinence | Unable to control the bladder so urine leaks out without control |
| Voluntary bowel movements | Whether the bowels work without needing enemas (liquid medicine given via the bottom), washouts (water or saline given via the bottom to washout poo) or a stoma (A stoma is where the bowel is joined to the skin on the tummy wall so that poo goes into a bag on the tummy as opposed to through the bottom). |

## Supplementary material two - Outcome modification, addition and dropping prior to consensus meeting

### Outcomes added by SMG prior to phase one (5)

- Time to full feeds
- Painful defecation
- Post-operative experience of pain
- Incisional hernia
- Stomal stenosis

### Outcomes split by SMG prior to phase one (3 split to 7)

- **“Readmission”** split to “**unplanned readmission”**, and “**planned re-admission”.**
- The outcome **“narrowing of anastomosis or cuff”** was felt to represent two different entities, and was therefore split into **“narrowing of anastomosis”**, and **“narrowing of cuff”**.
- “Peri-anal excoriation” was split into **“peri-anal excoriation with significant impact on daily life”**, **“peri-anal excoriation without significant impact on daily life”, and** **“peri-stomal excoriation with significant impact on daily life”,** and **“peri-stomal excoriation without significant impact on daily life”.**

### Outcomes modified by SMG prior to phase one (3)

- The steering committee felt that it was important that the outcome “**wearing a nappy”** also captured information on those requiring pads to maintain cleanliness. This outcome was therefore re-worded to “**wearing a nappy or pad”**
- It was felt that the outcome “**ICU admission”** should be expanded to “**ICU or HDU admission**”.
- The outcome **“Time of antibiotic administration**” was replaced with **“duration of antibiotic use post-operatively”**

### Outcomes dropped by SMG prior to phase one (4)

- The steering committee felt that the outcome **consistency of stool** would collect information on whether people thought that diarrhoea and constipation were important, as these represented extreme ends of a spectrum represented by consistency of stool. Other measures of diarrhoea and constipation would be captured by the outcomes frequency of defecation, and faecal incontinence. The outcomes **“constipation”** and **“diarrhoea”** were therefore removed.
- The outcome “**encopresis”** was felt to be too close to “**faecal incontinence**” and was therefore removed.
- The outcome **“anal lacerations”** was felt to be an outcome that represented poor surgical practice as opposed to one that would routinely be used to measure operative outcome and was therefore felt to be outside of the scope of this core outcome set. This outcome was therefore removed.
- The outcome **“necrosis and retraction of the colon”** was felt to be covered by the outcomes “**ischaemic bowel”** and **“anastomotic breakdown”**, and was therefore removed.

### Outcomes merged by the SMG prior to phase one (9 merged to 4)

- The steering committee members felt that the five outcomes “**long-term bowel dysfunction”, “assessment of bowel function”, “bowel function score”, “gastrointestinal quality of life index score”,** and **“Krickenbeck** **score”** needed to be revised. It was felt that the specific scoring systems would not have sufficient meaning for either clinicians or lay members to be accurately rated. It was felt that the important distinction to be made was whether people felt it important to have a unifying objective score that rated bowel function, a unifying objective score that rated quality of life, or an assessment of the individual components of the score. It was therefore determined that these five outcomes should be replaced with ***“Objective score of bowel function”*** and “***objective score of quality of life”.*** It was felt that the individual components of these scores would be captured in other outcomes such as consistency of stool, frequency of defecation etc.
- The outcomes “**early or persistent obstruction”**, and “**late or adhesional obstruction”** were felt to be too difficult to separate reliably by clinicians and lay members, and were therefore merged into one common outcome of “**post-operative bowel obstruction”.**
- The outcomes **“feeding intolerance”** and **“diet tolerated”** were felt to both be asking about long-term food intolerance, and were therefore merged to the common term “**long-term difficulty feeding or intolerance of food**”. Short-term feeding measures were felt likely to be captured by the outcomes including “**time to first feed”** and **“time to full feeds**”.

### Outcomes added between phase one and phase two (28)

- Lifetime healthcare costs
- Ability to control flatus
- Long-term offensive odour secondary to lack of control of faeces, or flatus, or inability to maintain hygiene
- Intra-operative complication - minor impact on recovery (Clavien-Dindo grade one)
- Intra-operative complication - requires additional medication (Clavien-Dindo grade two)
- Intra-operative complication - requires additional surgery (Clavien-Dindo grade three)
- Intra-operative complication - life threatening (Clavien-Dindo grade four)
- Intra-operative complication resulting in death (Clavien-Dindo grade five
- Intra-operative visceral injury - minor impact on recovery (Clavien-Dindo grade one)
- Intra-operative visceral injury - requires additional medication (Clavien-Dindo grade two)
- Intra-operative visceral injury - requires additional surgery (Clavien-Dindo grade three)
- Intra-operative visceral injury - life threatening (Clavien-Dindo grade four)
- Intra-operative visceral injury resulting in death (Clavien-Dindo grade five)
- Long-term need for dietary supplementation, or specialist exclusion diet
- Whether home parenteral nutrition is required
- Post-operative complication - minor impact on recovery (Clavien-Dindo grade one)
- Post-operative complication - requires additional medication (Clavien-Dindo grade two)
- Post-operative complication - requires additional surgery (Clavien-Dindo grade three)
- Post-operative complication - life threatening (Clavien-Dindo grade four)
- Post-operative complication resulting in death
- Any stoma morbidity requiring surgical intervention
- Need for a long-term or permanent stoma
- Need for a new stoma at any point after the pull-through procedure
- Difficulty in conceiving a child
- Difficulty with sexual relations or sexual intercourse, because of the psychological or physical impact of HD or its treatment.
- Regular post-operative anal sphincter Botox injections
- Attendance at school, or time spent missing lessons
- Normal growth

### Outcomes modified between phase one and two based upon feedback from phase one (19):

- Bladder Dysfunction 🡪 Long-term bladder dysfunction
- Cost 🡪 Operative cost
- Dehiscence 🡪 Wound dehiscence
- Depression 🡪 One or more periods of depression requiring treatment, at any age, as a result of the physical or psychological impact of the HD or its treatment
- Dilations 🡪 Need for anal dilations (other than a single post-operative calibration)
- Duration of post-operative antibiotic use 🡪 Duration of therapeutic, not prophylactic antibiotic use post-operatively
- Faecal incontinence 🡪 Long-term faecal incontinence
- Granuloma 🡪 Granuloma/excessive granulation tissue formation
- Intra-operative complication 🡪 *Any* intra-operative complication
- Intra-operative visceral injury 🡪 *Any* intra-operative visceral injury
- Long-term difficulty in feeding, or intolerance of food 🡪 Long-term difficulty in feeding
- Objective score of quality of life 🡪 Objective score of quality of life, using appropriate age specific measures
- Parenteral nutrition use 🡪 Duration of parenteral nutrition use
- Post-operative complications 🡪 *Any* post-operative complication
- Psychological stress 🡪 Long-term psychological stress
- Stoma morbidity 🡪 Any stoma morbidity that results in the child requiring additional pharmacological interventions
- Time to normal bowel habits 🡪 Time from operation to development of bowel habits that would be considered normal for someone without HD
- Urinary incontinence 🡪 Long-term urinary incontinence
- Voluntary bowel movements 🡪 Long-term voluntary bowel movements without need for enemas, peristeen, washouts or an ACE.

### Outcomes dropped between phase two and phase three (13)

- Whether an operation that started as a keyhole operation has to instead be performed through a bigger wound
- How much the operation cost (regardless of who pays these costs)
- How long antibiotics were given for to treat an infection  after the operation
- Whether there is an abnormally high temperature after the operation
- How long it was between the operation and bowels first opening
- Abnormal excessive healing tissue around a wound
- How long the total hospital stay was after the operation
- Complication during the operation that has a minor impact on recovery
- Complication during the operation which requires additional medicines to be given to treat it
- How long the operation took
- A complication after the operation which has minimal impact on recovery
- Time between the operation and first being able to have milk/food by mouth
- Time between the operation and taking full feeds by mouth without any need for fluid or feed through a drip

### Outcomes dropped between phase three and consensus meeting (44)

- Regular post-operative anal sphincter Botox injections
- Need for anal dilations (other than a single post-operative calibration)
- Unplanned Readmission
- Analgesic use post-operatively
- ICU or HDU admission
- Lifetime healthcare costs
- Planned readmission (e.g. for anal dilatations)
- Long-term need for dietary supplementation, or specialist exclusion diet
- Consistency of stool
- Duration of parenteral nutrition use
- Laxative medications
- Cosmetic results
- Peri-anal Excoriation without significant impact on daily life
- Peri-stomal Excoriation without significant impact on daily life
- Early pelvic inflammation
- Time from operation to development of bowel habits that would be considered normal for someone without HD
- Any stoma morbidity requiring surgical intervention
- Abscesses
- Stomal stenosis
- Intra-operative blood loss
- Intra-operative visceral injury –minor impact on recovery (Clavien-Dindo grade one)
- Intra-operative visceral injury - requires additional medication (Clavien-Dindo grade two)
- Stomal retraction
- Incisional hernia
- Stomal Prolapse
- Parastomal Hernia
- Post-operative complication - requires additional medication (Clavien-Dindo grade two)
- Pneumonia
- Abdominal distension
- Ability to control flatus
- One or more periods of depression requiring treatment, at any age, as a result of the physical or psychological impact of the HD or its treatment
- Post-operative experience of pain
- Sleep disturbance
- Frequency of bowel movements
- Sphincter achalasia
- Post-operative complication - requires additional surgery (Clavien-Dindo grade three)
- Intra-operative visceral injury - requires additional surgery (Clavien-Dindo grade three)
- Any intra-operative complication
- Any post-operative complication
- Post-operative infection
- Adhesions
- Any stoma morbidity that results in the child requiring additional pharmacological interventions
- Anorectal Manometry
- Objective score of bowel function
